# Examining Vaccine Sentiment on Twitter and Local Vaccine Deployment during the COVID-19 Pandemic

**DOI:** 10.3390/ijerph20010354

**Published:** 2022-12-26

**Authors:** Lourdes S. Martinez, Matthew W. Savage, Elisabeth Jones, Elizabeth Mikita, Varun Yadav, Ming-Hsiang Tsou

**Affiliations:** 1 School of Communication, San Diego State University, San Diego, CA 92182, USA; 2 College of Arts and Letters, San Diego State University, San Diego, CA 92182, USA; 3 School of Public Health, San Diego State University, San Diego, CA 92182, USA; 4 Department of Biochemistry, University of California, San Diego, CA 92093, USA; 5 Department of Geography, San Diego State University, San Diego, CA 92182, USA

**Keywords:** vaccines, Twitter, Communication Infrastructure Theory, sentiment, COVID-19

## Abstract

Understanding local public attitudes toward receiving vaccines is vital to successful vaccine campaigns. Social media platforms may help uncover vaccine sentiments during infectious disease outbreaks at the local level, and whether offline local events support vaccine-promotion efforts. Communication Infrastructure Theory (CIT) served as a guiding framework for this case study of the San Diego region examining local public sentiment toward vaccines expressed on Twitter during the COVID-19 pandemic. We performed a sentiment analysis (including positivity and subjectivity) of 187,349 tweets gathered from May 2020 to March 2021, and examined how sentiment corresponded with local vaccine deployment. The months of November and December (52.9%) 2020 saw a majority of tweets expressing positive sentiment and coincided with announcements of offline local events signaling San Diego’s imminent deployment of COVID-19 vaccines. Across all months, tweets remained mostly objective (never falling below 63%). In terms of CIT, considering multiple levels of the Story Telling Network in online spaces, and examining sentiment about vaccines on Twitter may help scholars to explore the Communication Action Context, as well as cultivate positive community attitudes to improve the Field of Health Action regarding vaccines. Real-time analysis of local tweets during development and deployment of new vaccines may help monitor local public responses and guide promotion of immunizations in communities.

## 1. Introduction

In 2020, the United States (U.S.) reported 19,513,331 confirmed cases of the novel coronavirus that causes the coronavirus disease 2019 or COVID-19 (also known as severe acute respiratory syndrome coronavirus 2 or SARS-CoV-2) [1]. An estimated 345,398 COVID-19 deaths were reported during the same time frame in the U.S. [1]. COVID-19 vaccines (such as ones developed as a result of the pandemic) and their safe and equitable distribution [2] have been recognized as playing an essential part in ending the pandemic, restoring public health and recovering economic losses in the U.S. and across the globe [3].

By March 2021, three COVID-19 vaccines received emergency use authorization by the U.S. Food and Drug Administration (FDA). Although the Pfizer, Moderna, and Johnson & Johnson vaccines demonstrated effectiveness in protecting against severe cases of COVID-19 [4], vaccine distribution varied locally across different communities and challenges in rapid and equitable vaccine deployment soon surfaced [5]. Vaccine hesitancy emerged as a barrier to sufficient distributions of vaccines required to achieve herd immunity [6] and address health disparities magnified by the COVID-19 pandemic [7], especially in communities of color [8].

Given the importance of vaccines for ending the COVID-19 pandemic and future outbreaks of infectious diseases along with the lack of a comprehensive national strategy for addressing COVID-19 [9,10], more research is needed to understand public perceptions of vaccines at the local level. Public perceptions of vaccines at the local level warrant additional attention for a few reasons. First, as we have seen with COVID-19, public reception to vaccines can vary substantially across different U.S. regions and communities [11]. Second, local factors on the ground can affect the spread of infectious diseases like COVID-19 [12,13] and potentially whether local populations perceive a need for vaccines. Third, a lack of awareness regarding how a specific region or community views vaccines may present challenges to creating effective public health campaigns for vaccine promotion. Thus, the purpose of the present exploratory and descriptive study is to draw upon Communication Infrastructure Theory and examine the San Diego region of California as a case study to investigate local public sentiment toward vaccines expressed on Twitter during the COVID-19 pandemic, and how this sentiment corresponds with offline local vaccine events during the same time frame.

### 1.1. Public Attitudes toward Vaccines

Behavior change research guided by reasoned-action theorizing suggests that attitude toward vaccine uptake often operates as a key predictor of vaccine intentions [14,15,16] and behavioral outcomes [17,18] for a range of infectious diseases. Behavioral beliefs about vaccination uptake underlie attitudes toward vaccine uptake, and can be conceptualized as one’s beliefs surrounding the likelihood of outcomes resulting from vaccine uptake and whether one views these outcomes as favorable or unfavorable [19].

Although most individuals living in the U.S. currently hold a favorable view toward vaccines [20], evidence indicates a growing portion are now vaccine hesitant [21,22]. Vaccine hesitancy involves feelings of diminished trust in vaccines or health care providers, views that vaccination is unnecessary, and lack of convenient access [23]. Anti-vaccine activities drive reductions in herd immunity and are associated with recent re-emergence of vaccine-preventable diseases in regions like California [24,25]. At times, hesitancy can translate into outright refusal. Individuals who are vaccine hesitant may range from those who are unsure whether to accept vaccination to complete refusal [26].

Given the importance of political dimensions in shaping vaccine-related behavior and policy [27,28,29], this study examines San Diego as a case study for local vaccine sentiment. The intersection of vaccine hesitancy with American political ideologies is now receiving more scholarly attention [21,30,31], and the political diversity of San Diego makes it an excellent site for case study. That is, San Diego is the second largest city in California [32], with a more even share of Republicans and Democrats who voted in the last Presidential election than the largest city in the state, Los Angeles [33]. As a city in the state of California (which has emerged as a leader in recent vaccination legislation), San Diego is also uniquely positioned as a study context for examining vaccine sentiment and deployment given its local participation in vaccine development, where San Diego sites comprised three of the seven clinical trial sites for Moderna in the state of California [34].

### 1.2. Communication Infrastructure Theory

Explanations for vaccine hesitancy for individuals may be embedded within larger community perceptions of health. To account for this, a social ecological approach is employed here to account for the varied layered contexts that influence health attitudes and behavior. Communication infrastructure theory (CIT) is an ecological theory explaining the structure of health perceptions and decision-making as a function of communication systems, represented in connections between community members at multiple levels [35]. Scholars have drawn on CIT to study topics such as civic engagement, health disparities, and community-engaged health interventions [36,37,38,39], demonstrating that CIT offers a grounded theoretical framework for practical health promotion scholarship by accounting for the connection between individual actors and community level communication structures.

CIT explains that conditions at multiple levels (e.g., individual, interpersonal, organizational, community, and societal) makeup the *communication ecology* of specific geographic regions, and these influence health perceptions, goals, and decision-making [36,40]. A communication ecology is the network of communication resources upon which a person draws in enacting a behavior [41], such as getting vaccinated. Acknowledging communication ecologies underscores that the message exchange of community members occurs within the context of larger communication systems, and that the shared perceptions and attitudes which emerge can have implications for community health [42]. Further, CIT goes on to describe an interactive relationship between communication ecologies within the *storytelling network* (STN) and the *communication action context* (CAC). Together, the STN and CAC dynamically reveal the *field of health action* (FHA).

A STN exists at the macro, meso, and micro levels, based on the storyteller and audience, and recent research shows how a STN can exist within social media [43]. Examining communication on Twitter allows us to delve into the digital layers of the neighborhood storytelling network. For example, scholars [44] determined that social networking sites provide a platform for storytellers to extend their interactions and connect to form a STN. Linkages are established through online connections, and in the present study we extend this analysis to consider perceptions and attitudes within the STN. Indeed, the CAC allows for this to take place.

The CAC refers to the built and social environment that enables or constrains storytelling based on communication resources within a community [41], and recent scholarship recognizes social media as part of the CAC. Nah et al. [45] extended CIT to assert that social media is, “an important storytelling agent just like traditional media or community organizations in the digitally mediated civic communication environment, as they help circulate community-bound news information, promote interaction and dialogue among fellow residents, and serve as the gateway connecting residents and local community organizations” (p. 5). The CAC recognizes how personal and social environments become integrated, and when it comes to health outcomes, CIT argues that the interplay of the STN and the CAC reveal a community’s FHA.

The FHA is the “sociomaterial context that comprises a place-specific set of structural conditions and interpretive resources, within which residents may be more or less inclined to seek particular kinds of health-care services and respond favorably to a health-promotion intervention” [36] (p. 168). FHAs are not just the physical places that healthcare is enacted, but also the subjective meanings about health topics that community members determine through shared understanding. In this way, the FHA is dynamic and changing based on the STN and CAC. A communication tool that impacts what community members believe about the FHA related to vaccines is Twitter.

### 1.3. Social Media & Sentiment Analysis

Social media use occupies a large part of the daily lives of many Americans and can impact health outcomes. For example, an estimated seven in ten Americans report using any social media [46]. In addition, 53% of U.S. adults note they use social media “sometimes” or “often” as a news source [47]. Although concerns regarding accuracy of news on social media have risen [48,49,50], users continue to consume news shared on social media. In particular, Twitter is a highly consulted source of news, with 71% of Twitter users reporting they obtain their news on the platform [51]. Furthermore, research recognizes the potential for social media to impact health contexts, including vaccine acceptance [52]. Given unique properties of social media allowing users to purposefully share content and possibly ignite its propagation across social networks, it is important to continue examining vaccine-related content shared on social media about vaccine attitudes and behavior.

One way to examine vaccine attitudes on social media is through sentiment analysis. Sentiment analysis, or opinion mining, is an approach that “analyzes people’s opinion, sentiment, evaluation, appraisal, attitude, and emotion towards entities such as product, service, organization, individual, issue, event, topic, and their attributes” [53] (p. 4). The terms sentiment analysis [54] and opinion mining [55] predate social networking sites such as Twitter, however the social media analytical techniques they inspired are widely used in current Twitter vaccine research [56,57,58,59]. Sentiment analysis, as a technique tapping into an evaluative dimension of attitude, can provide some indication of public attitudes toward vaccines expressed on social media by examining properties of such content, including polarity and subjectivity. Polarity refers to the degree to which content is expressed in a positive or negative tone, while subjectivity captures the extent to such content is characterized by more objective or subjective features [60].

### 1.4. Purpose of the Study & Research Questions

Twitter provides an online communication ecology revealing sentiments about vaccines in particular localities warranting investigation. Through the lens of CIT, vaccine adoption or hesitancy may be affected by the perception of other resources in the CAC that are revealed on Twitter. Further, perceived characteristics of the local STN can impact perceptions of barriers and facilitators to vaccine uptake based on normative perceptions shared in Tweets by family and friends, colleagues and coworkers, opinion leaders, news sources, and the like across multiple levels of influence within an individual’s life. Community attitudes toward vaccines can be understood as the product of a multidimensional relationship between the CAC and the STN. Therefore, in the present case study, frequency, polarity, and subjectivity of vaccine-related tweets in the San Diego region are investigated to explore important community-level aspects of the FHA concerning vaccines. Thus, the following research questions are offered:

RQ1: What was the (a) frequency of vaccine-related tweets during the COVID-19 pandemic and (b) how did it vary over time?
RQ2: What was the (a) local polarity of vaccine-related tweets expressed during the COVID-19 pandemic and (b) how did it vary over time?
RQ3: What was the (a) local subjectivity of vaccine-related tweets expressed during the COVID-19 pandemic and (b) how did it vary over time?

### 1.5. Local Vaccine Deployment & Research Question

In the U.S., several political and public health responses to the COVID-19 pandemic have been largely shaped at the local level [61]. For example, the state of California defined tiers [62] for when different groups became eligible for COVID-19 vaccination and messaging about these tiers was shared through mass media at the macro level. Each county shared messaging about its own timeline [63] for distributing COVID-19 vaccinations once they became available to their communities [62]. Hospitalizations and deaths varied so much from one community to another [64], stories about the locale regarding important offline events related to vaccines were likely distributed at the meso level by regional media and community organizations too. These contributions to the STN may have impacted vaccine-related sentiment expressed on Twitter in San Diego during the vaccine distribution timeframe. To explore the interplay of communication at multiple levels in this case study, we examined a final question:

RQ4: Did local (a) polarity and (b) subjectivity expressed on Twitter correspond with vaccine deployment during the COVID-19 pandemic?

## 2. Methods

### 2.1. Sample

From May 2020 to March 2021, a total of 314,851 geocoded tweets in the San Diego region mentioning vaccines were collected. Unlike geo-tagged tweets which already include location coordinates and only capture 1% of total tweets [65], our method for geocoding locations for tweets in the present study relied on place-name dictionaries (gazetteers) available in user profile information. This approach can geocode about 50% of total available tweets [66,67].

### 2.2. Data Collection

#### 2.2.1. Twitter Data

We used software known as the Social Media Analytic and Research Testbed (SMART) dashboard [68] to gather vaccine-related tweets. This software uses a social media application program interface (API) in tandem with geographical information system (GIS) technology. Features of the SMART dashboard permit users to create visualizations of topics trending on Twitter [69] at various user-defined geographical layers, ranging from local to global levels. Users can then denote specific topics defined by keyword inputs. When these keywords are entered into the SMART dashboard, tweets that contain the specified keywords are filtered using a Twitter Search API and collected daily. As part of these daily collections, the Twitter Search API uses an accumulative method in which each day’s collection is compared to the prior day’s collection, and only new collections are retained in databases. Using keywords inputs, the SMART dashboard is usually able to continuously gather tweets without gaps in data collection due to capabilities of Twitter’s Search APIs which can gather close to 18,000 tweets every 15 min. All data gathered are then stored in the MongoDB database and from which visualizations can be generated using the dashboard.

To identify appropriate inputs for the dashboard and ensure relevant tweets were gathered, for this study we selected general key words adapted from other investigations of vaccine perceptions on social media [70,71]: “vaccine”, “vaccination”, “vaxxer”, “vaxx”, and “vacc”. Inclusion criteria for this study required that each tweet gathered for analysis (1) included at least one of these keywords and (2) was written in the English language.

#### 2.2.2. Significant Offline Events: Local Vaccine Deployment

As the COVID-19 pandemic touched almost every aspect of life during the time period in which we collected data, we focused on significant offline events most relevant to COVID-19 vaccines that received either local or national news coverage. For this reason, we used the initiation of vaccination tiers and timeline for their deployment in San Diego as appropriate to represent the focus of this case study. Tiers included in this analysis were Tier 1A beginning on 15 December 2020 (e.g., emergency, inpatient, outpatient, and community health workers), Tier 1B beginning on 27 February 2021 (e.g., individuals aged 65+, workers in essential services sectors, and those working in other emergency services sectors), and Tier 1C beginning on 15 March 2021 (e.g., individuals with certain medical conditions, the homeless, and transit workers).

### 2.3. Analytical Approach & Sentiment Analysis

Our analytical approach for this study was largely descriptive. In addition to performing basic frequency distributions, analyses involved overlaying data from Twitter and significant offline events in order to determine whether certain variables corresponded with others and to uncover patterns that unfolded over time. Below we also describe our approach to sentiment analysis, which allowed us to conduct analyses with variables capturing polarity and subjectivity. Prior to analysis, data were cleaned to remove nonsensical and gibberish responses. Our data cleaning process consisted of removing HTML special entities (e.g., &amp), hyperlinks, ticker symbols, removing words with 2 or fewer characters, white spaces, and any characters beyond basic multilingual plane of Unicode. We also removed all bots prior to performing any analysis using a white list method described in prior research [72]. This approach allowed us to retain tweets from reliable actors at all levels of the STN, and reduced our analytical sample to 11,324 tweets.

We performed a sentiment analysis of vaccine-related tweets in order to assess an evaluative dimension of attitude toward vaccines expressed in each tweet gathered for this study. One key feature of sentiment analysis is that it is able to classify Tweet text as emotional, opinionate, or idea-based content. We used the TextBlob Library in Python [73], a common lexicon used for sentiment analysis that has been used in similar research [74] and is on par with related lexicons. A lexicon essentially provides a “dictionary” of words that are commonly associated with positive, neutral, and negative sentiment. As part of a sentiment analysis, Textblob can generate scores for polarity and subjectivity. Scores for polarity can range from −1.00 to 1.00, with a score above 0.00 denoting positivity and below 0.00 denoting negativity in tone. In contrast, subjectivity can only score anywhere between 0.00 and 1.00. A subjectivity score closer to 1.00 suggests more subjectivity (e.g., “I hate getting side effects from vaccines”) while a score closer to 0.00 indicated more objectivity (e.g., “vaccines do not cause serious side effects for most people”). As Textblob allows for basic level sentiment analysis, this lexicon is appropriate for the scope of the present research given the exploratory nature and the unique effort of this study to show patterns of vaccine sentiment expressed about local vaccine deployment on Twitter.

## 3. Results

### 3.1. Frequencies and Over-Time Variations (RQ1–RQ3)

**Figure 1:**
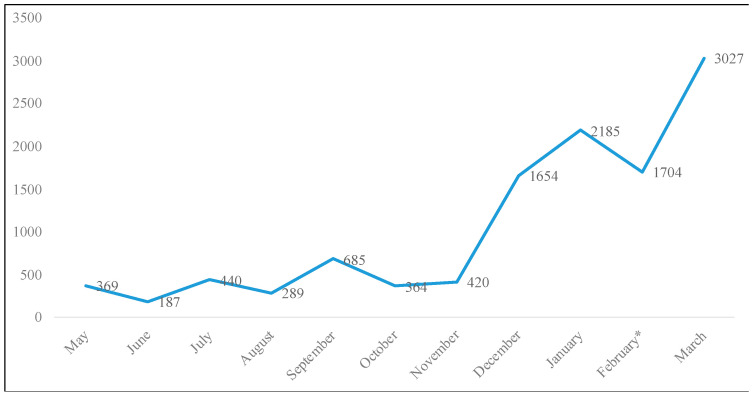
Number of tweets regarding the vaccine keyword over time. * Note: February data collection only includes data from 1–19 February.

Our first research question sought to determine the frequency of vaccine-related tweets (RQ1a) during the COVID-19 pandemic and how it varied over time (RQ1b). Descriptive statistics were performed first to understand baseline levels of vaccine discussion within San Diego’s tweets. Results displayed in Figure 1 showed that from May 2020 to March 2021, there was a great variability of discussion on vaccines. The lowest amount of discussion during the timeframe occurred in June of 2020 with 187 tweets regarding the vaccine. The discussion on vaccines reached a peak in March of 2021 with a total of 3027 tweets.

**Figure 2:**
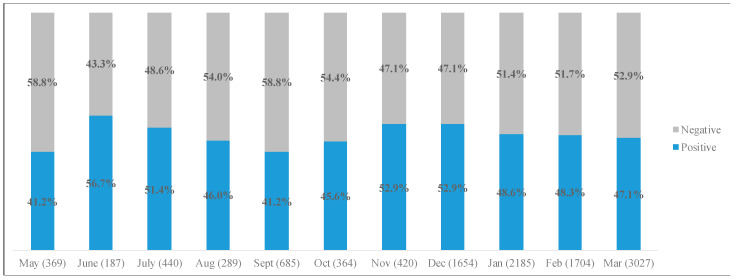
Positivity of tweets by month.

To answer the second research question, sentiment analyses were conducted by month to determine frequency (RQ2a) and polarity (expressed as overall positivity) across the timeframe (RQ2b). Results of this analysis are presented in Figure 2. Regarding positivity of tweets, the months with the highest amount of positive sentiment were June (56.7%), November and December 2020 (52.9% positive), and July 2020 (51.4% positive).

**Figure 3:**
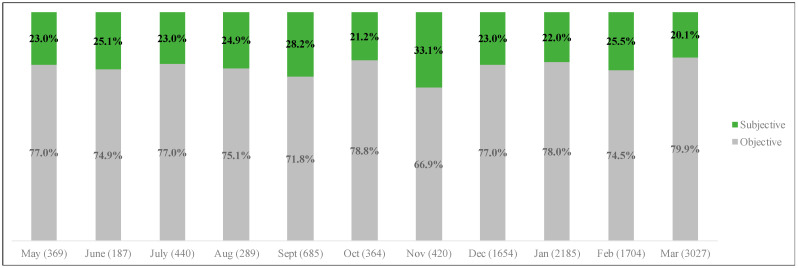
Subjectivity of tweets by month.

Turning now toward subjectivity, our third research question sought to establish frequency (RQ3a) and overtime variations in subjectivity expressed within tweets (RQ3b). Analysis of frequency and overtime variations in subjectivity of tweets are illustrated in Figure 3. Results showed that across all months, there were higher amounts of objective tweets than subjective tweets. In other words, most tweets contained factual content rather than subjective expressions of opinions in every month. November 2020 showed the highest amount of subjective tweets (33.1%), followed by September 2020 (28.2%). The months with the highest amount of objective tweets included March 2021 (79.9%), October 2020 (78.8%), and January 2021 (78.0%).

### 3.2. Correspondence with Local Vaccine Deployment (RQ4)

**Figure 4:**
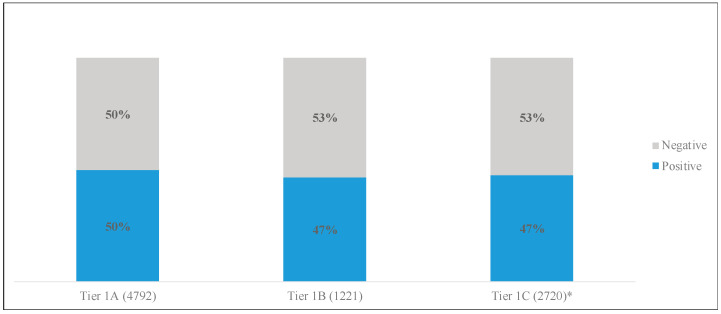
Positivity of tweets by vaccination tier. * For these analyses Tier 1C includes data from 15–31 March 2021.

To address the fourth research question, polarity (expressed as overall positivity) (RQ4a) and subjectivity (RQ4b) of vaccine-related tweets were compared to the occurrence of deployment of vaccines within the timeframe of San Diego County’s vaccination tiers. Positive sentiment varied slightly between the tiers with Tier 1A showing the highest positive sentiment at 50%. Tier 1B and 1C displayed the lower amounts of positivity with 47% classified as positive tweets and 53% as negative. Figure 4 displays these results in greater detail.

**Figure 5:**
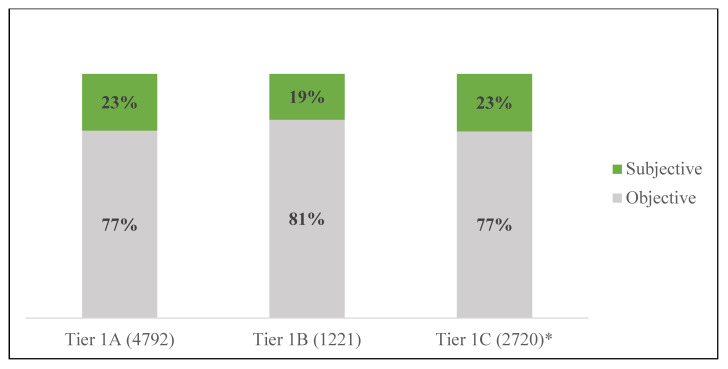
Subjectivity of tweets by vaccination tier. * For these analyses Tier 1C includes data from 15–31 March 2021.

We repeated this same procedure to examine if subjectivity varied across vaccination tiers. All three tiers displayed higher amounts of objective tweets than subjective. According to Figure 5, Tiers 1A and 1C contained the highest amounts of subjective tweets (23% subjective and 77% objective). The lowest amount of subjective tweets was observed during the deployment of vaccines in Tier 1B (19% subjective and 81% objective).

## 4. Discussion

Using San Diego as a case study, this study describes the level of local attention and sentiment toward vaccines during the COVID-19 pandemic. From May 2020 to March 2021, we observed fluctuations in the local attention given to vaccines on Twitter, with the highest level of attention occurring in March 2021, the same month that saw deployment of vaccines to eligible residents in Tier 1C in San Diego. The rise in attention toward vaccines began in December 2020. During this timeframe, we also observed variability with polarity where the level of positivity toward vaccines peaked in November and December 2020, and corresponded with initiation of local COVID-19 vaccine deployment. The rise in attention and peak in positivity both occurred within the same month as the local start of vaccine distribution. This is consistent with prior research showing new vaccines can attract significant media attention—especially when shrouded in controversy [75,76]. These findings are also consistent with research suggesting communities may be more accepting, receptive, and favorably responsive to interventions that are perceived to be developed with a community-centered model [77]. Specifically, the participation of local clinical trial sites such as La Jolla, La Mesa, and San Diego in Moderna vaccine trials [34] may have helped boost positivity and hope that COVID-19 vaccines would help bring a swift end to the pandemic [78].

However, positivity later dropped off after implementing vaccination tiers for eligible groups. In fact, positivity was at its highest at 50% during the rollout of vaccines in Tier 1A, before dropping and staying at 47% during the rollout of vaccines in Tier 1B and 1C. One explanation for this could be that the conversation shifted from one characterized by vaccine optimism and an imminent return to pre-pandemic life toward one driven by stories discussing logistical challenges with vaccine deployment [79] and adverse vaccine effects [80]. This finding is important because it suggests that the deployment of vaccines may draw attention to vaccines and represent a key window to monitor the information environment, engage in efforts to keep vaccine sentiment positive, and quickly identify misinformation and correct misperceptions.

Although we observed variability in polarity throughout our study’s timeframe in the COVID-19 pandemic, subjectivity did not appear to fluctuate as much and never described more than 33.1% of the vaccine-related tweets in any given month within our study. Even when we examined the implementation of vaccine tiers, the proportion of vaccine-related tweets classified as objective remained dominant. This could have been the result of high levels of uncertainty that characterized the information environment during the COVID-19 pandemic [81]. In an information environment rife with uncertainty regarding characteristics of COVID-19 and new local policies governing daily life [82], those in the STN may have been more focused on gathering and possibly sharing content that contained more factual information about what was currently understood about COVID-19 (e.g., new local cases) and local responses (e.g., information on mask mandates) rather than opining.

### 4.1. Theoretical and Practical Implications

This study has a number of implications for theory and practice in health communication. First, these findings may be useful for CIT theorists. Our study illustrates a co-occurrence of sentiment expressed on Twitter and regional vaccine rollout, revealing an attentional dynamic by individuals who are interfacing in the CAC with actors at multiple levels of the STN. In this way, community sentiment represents a product of a dynamic relationship between the STN and the CAC, and this transaction reveals the FHA where residents become more or less likely to get vaccinated. Scholars are recognizing the importance of online communication ecologies and how social media impacts the STN [44], and our research extends arguments supporting the notion of integrated connectedness to a STN [83]. Those employing CIT might consider using sentiment analysis to empirically investigate the FHA by investigating important digital tools in the CAC. Measuring sentiment may reveal underlying community-level attitudes, which could be done in conjunction with (or even when it is not possible to get) individual-level data from community members measuring neighborhood belonging, collective efficacy, or civic participation. These are variables central to CIT scholarship that researchers in recent studies have begun to consider online, described as integrated connectedness to the STN [83,84]. Expanding social media research using sentiment analysis in different places, cultures, and communities can help to understand the FHA in a locality. This is important because public health responses can vary across populations and communities.

Second, CIT provides key practical recommendations for vaccine health campaigns and interventions. CIT emphasizes the role of multiple levels of influence within an ecological framework that account for individual, interpersonal, organizational, community, and societal influences on health decision making. Integrating considerations from each level into the persuasive message design of local public health promotion campaigns disseminated on social media may improve campaign effects. Introducing persuasive vaccine promotion messages to Twitter may be fruitful when done in conjunction with the timing other community-based vaccine promotion strategies. Twitter may be a rewarding space for health communication scholars to join the conversation, alongside community organizations and locally based media. Described as *geo-ethnic media* because they focus on reaching particular geographic locations or local ethnic groups [85], these locally based media are able to “mobilize residents around community issues and the broader realm of civic life” [86] (p. 329). Twitter can be an important tool in the CAC which scholars can explore to improve community attitudes about vaccines. Health campaigners must meet communities where they are in saturated social media landscapes to enhance factual information-sharing about vaccines, for the purpose of bolstering the vaccine FHA. Doing so can help improve perceptions of vaccines as an accessible and effective public health priority.

### 4.2. Limitations and Future Directions

The purpose of this study was to probe if any potentially interesting patterns between deployment of new vaccines and sentiment expressed in vaccine-related tweets emerged. The study however is limited in that it is not correlational in nature and we do not precisely analyze correlations between these events and tweet sentiment. To the best of our knowledge, this study is among the first of its kind to pursue questions of tweet sentiment and offline events related to COVID-19 vaccines and represents a first step for exploration in this area. Our study suggests patterns that warrant further exploration and a fertile area for future research.

Other additional limitations of this study warrant discussion. First, this study only examines vaccine-related tweets on Twitter written in English. The results of this study may not generalize to sentiments regarding other public health issues, vaccine sentiments shared on other social media platforms, or those written in languages other than English. Second, the keywords used to collect vaccine-related tweets may not have been exhaustive. For example, we did not include “shot” as a keyword although often used interchangeably with “vaccine”. Third, sentiment analysis is limited in addressing nuances in language use and the role of context when classifying sentiment of social media content. Our study is also limited by its use of tweets, the content of which is not always credible. We also did not assess the credibility of the posts as to whether they contained any misinformation. There is also a selection bias in examining tweets given that not everyone is a Twitter user, and not every Twitter user shares tweets containing vaccine-related content. In this manner, the usefulness of sentiment analysis as a measure for gauging where and how to direct prevention efforts in the context of emerging infectious diseases is also limited. An additional limitation relates to our data cleaning process which could evoke an artificial bias in tweets that were excluded and thus affecting our results. Last, because the goal of this study was to be exploratory we did not take into account multiple levels of analysis and only focused on conducting a descriptive case study of vaccine deployment in one region where empirical data was limited to the micro-level. Although this is a limitation, it is in line with a range of other published CIT scholarship that empirically draws from one level of the STN but makes conceptual connections across the CAC and FHA [36,38,87]. Future scholars could address this limitation by designing studies that capture within and between ecological levels, such that multilevel modeling approaches can be employed to analyze these complexities over time. Further, time series analysis could be used to investigate how significant offline events across different regions are related to patterns of Twitter sentiment as health events unfold and impact communities.

We also encourage future scholars to employ other approaches to address additional limitations beyond the scope of the study. As we did not include a bi-directional sentiment analysis, future scholars may consider using other machine learning approaches such as BERT [88]. Additionally, we were unable to indicate if sentiment in tweets were specifically about “vaccine” or other content, however this could be examined in future research using supervised or semi-supervise sentiment analysis. Scholars may examine other social media platforms and content shared in different languages to verify persistence of patterns observed here. In addition, future researchers may explore whether patterns here apply to other public health contexts by examining, for example, sentiment toward wearing masks to prevent COVID-19 and if or how this sentiment corresponded with local events regarding mask-wearing policies.

Despite these limitations, this study is among the first to use geocoded tweets to explore the co-occurrence of local vaccine sentiment on Twitter with local vaccine deployment during a global pandemic. The use of geocoded tweets remains a fairly new approach for gaining understanding of local environments that may offer deeper insights for crafting more targeted community-based health intervention strategies [89]. Although a growing literature examining vaccine sentiment using geolocation data is developing [56,90], the present study contributes to this body of work by providing analyses of polarity and subjectivity at the local level.

## 5. Conclusions

Public health professionals may find value in these findings, specifically when designing and monitoring ongoing interventions. Communicating local involvement in vaccine development and deployment as part of a campaign strategy may help cultivate positive local sentiments toward vaccines, which may translate into higher rates of immunizations that protect herd immunity. Additionally, tools using real-time analysis of local tweets may be developed to track local events to help public health professionals monitor local public responses to efforts promoting immunizations in communities, to allow for adjustment to ongoing campaigns based on real-time community needs.
